# Supplementary material for: Sustained-input switches for transcription factors and microRNAs are central building blocks of eukaryotic gene circuits
Source: Genome Biol. 2013 Aug 23;14(8):R85. doi: 10.1186/gb-2013-14-8-r85 (PMC4054853; doi:10.1186/gb-2013-14-8-r85)
Supplement: Additional file 5 — HTML Browsable Motif Output. Zipped folder containing all WaRSwap and FANMOD motif output, viewable in a web browser. [file gb-2013-14-8-r85-S5.ZIP › HTML_browsable_motif_output/WaRSwap_ath_tair9/sigs_WSWRSLDraw_2-2000.pvals.heatmaps.html/motif_id_36_001001002_tftype_ath_upstream_-2000_0.html]

```
BG_MODEL = Rnew2
MOTIF_ID = 36_001001002
TF_TYPE = ath
UPSTREAM = -2000_0


PVals
FN_0.2	FN_0.4	FN_0.6	FN_0.8
dg_60.genes	0.384	0.638	0.999	0.001
dg_70.genes	0.391	0.62	1	0.002
dg_80.genes	0.407	0.609	1	0.001

ZScores
FN_0.2	FN_0.4	FN_0.6	FN_0.8
dg_60.genes	0.272	-0.41	-2.663	2.582
dg_70.genes	0.263	-0.346	-2.722	2.688
dg_80.genes	0.219	-0.35	-2.767	2.634

StDevs
FN_0.2	FN_0.4	FN_0.6	FN_0.8
dg_60.genes	29482.208	33629.162	13985.487	2963.759
dg_70.genes	30198.197	33221.422	13848.96	2890.978
dg_80.genes	29770.346	34158.397	13794.776	2925.635
```
